# CD34 ^+^ cell therapy significantly reduces adverse cardiac events, health care expenditures, and mortality in patients with refractory angina

**DOI:** 10.1002/sctm.20-0046

**Published:** 2020-06-12

**Authors:** Grace L. Johnson, Timothy D. Henry, Thomas J. Povsic, Douglas W. Losordo, Ross F. Garberich, Larissa I. Stanberry, Craig E. Strauss, Jay H. Traverse

**Affiliations:** ^1^ Minneapolis Heart Institute Foundation Abbott Northwestern Hospital Minneapolis Minnesota USA; ^2^ Cardiovascular Division The Christ Hospital Cincinnati Ohio USA; ^3^ Division of Cardiology Duke University School of Medicine Durham North Carolina USA; ^4^ Caladrius Biosciences Rye Brook New York USA; ^5^ Cardiovascular Division University of Minnesota School of Medicine Minneapolis Minnesota USA

**Keywords:** CD34^+^ stem cells, cost analysis, major adverse cardiac events, refractory angina

## Abstract

Patients with refractory angina who are suboptimal candidates for further revascularization have improved exercise time, decreased angina frequency, and reduced major adverse cardiac events with intramyocardial delivery of CD34^+^ cells. However, the effect of CD34^+^ cell therapy on health care expenditures before and after treatment is unknown. We determined the effect of CD34^+^ cell therapy on cardiac‐related hospital visits and costs during the 12 months following stem cell injection compared with the 12 months prior to injection. Cardiac‐related hospital admissions and procedures were retrospectively tabulated for patients enrolled at one site in one of three double‐blinded, placebo‐controlled CD34^+^ trials in the 12 months before and after intramyocardial injections of CD34^+^ cells vs placebo. Fifty‐six patients were randomized to CD34^+^ cell therapy (n = 37) vs placebo (n = 19). Patients randomized to cell therapy experienced 1.57 ± 1.39 cardiac‐related hospital visits 12 months before injection, compared with 0.78 ± 1.90 hospital visits 12 months after injection, which was associated with a 62% cost reduction translating to an average savings of $5500 per cell therapy patient. Patients in the placebo group also demonstrated a reduction in cardiac‐related hospital events and costs, although to a lesser degree than the CD34^+^ group. Through 1 January 2019, 24% of CD34^+^ subjects died at an average of 6.5 ± 2.4 years after enrollment, whereas 47% of placebo patients died at an average of 3.7 ± 1.9 years after enrollment. In conclusion, CD34^+^ cell therapy for subjects with refractory angina is associated with improved mortality and a reduction in hospital visits and expenditures for cardiac procedures in the year following treatment.

## Graphical abstract

The total cardiovascular hospitalizations, procedures and emergency room visits are significantly reduced in patients with refractory angina in the 12 months following intramyocardial CD34^+^ progenitor cell injections compared to the 12 months prior to injections (*P* = .002).

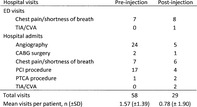

1

**Lessons learned**

- Patients with refractory angina who underwent intramyocardial injections of CD34+ stem cells had reduced hospitalizations, cardiac procedures and health care expenditures in the year following treatment compared to the year prior.
- CD34+ cell therapy significantly reduced mortality compared to placebo in patients with refractory angina.

**Significance statement**

Intramyocardial delivery of CD34^+^ progenitor cells to patients with refractory angina who are not candidates for further revascularization improves exercise time, decreases angina frequency, and reduces major adverse cardiac events. This report demonstrates for the first time that patients who received these cells experienced reduced health care utilization and costs in the year following therapy compared with the year before their administration. Furthermore, patients who received CD34^+^ cell therapy showed reduced mortality compared with patients who received placebo.

## 2 INTRODUCTION

As mortality from coronary artery disease declines, a growing number of patients experience refractory angina each year.^1^ The European Society of Cardiology has defined refractory angina as “a chronic condition characterized by the presence of angina caused by coronary insufficiency in the presence of coronary artery disease which cannot be controlled by a combination of medical therapy, angioplasty, and coronary bypass surgery.”^2^ Patients with refractory angina are often ineligible for further surgical revascularization owing to comorbidities or advanced age with suboptimal coronary anatomy due to diffuse disease or chronic total occlusions.^3^ The prevalence of refractory angina is unclear, with estimates varying from 300 000 to 1.7 million patients in the United States^1^, ^4^ and up to 200 000 new cases per year.^5^ Because of the poor quality of life^6^ and high resource use^7^ associated with this patient population, the development of effective and cost‐efficient therapies is critical.

There has been ongoing interest in therapeutic angiogenesis as a method of improving cardiac perfusion.^3^, ^8^ Preclinical studies have shown that intramyocardial transplantation of progenitor cells into ischemic tissue stimulates blood vessel growth.^9^, ^10^ In particular, cells expressing the surface protein CD34^+^ have demonstrated heightened efficacy in promoting neovascularization of myocardium.^11^ Strong preclinical data led to the development and implementation of three double‐blind, placebo‐controlled trials of CD34^+^ cells with similar designs.^12^, ^13^, ^14^, ^15^ Recently, a patient‐level meta‐analysis of these trials demonstrated a significant improvement in exercise time, a reduction in angina and in major adverse cardiac events,^15^ and an improvement in exercise capacity compared with subjects randomized to placebo.^15^

Despite these promising results, the effect of CD34^+^ cell therapy on cardiac‐related events, hospitalizations, and costs is unknown. Although these patients are believed to have high health care costs, investigation into their health care expenditures is limited.^16^ Therefore, we compared the number of cardiac hospital admissions and cardiovascular procedures in the 12 months prior to treatment vs the 12 months after treatment for patients randomized to injection of autologous CD34^+^ cells compared with placebo. We then conducted a cost analysis to determine whether CD34^+^ cell therapy was associated with a reduction in hospital expenditures within the first year after treatment.

## 3 MATERIALS AND METHODS

A retrospective chart review was performed for patients at The Minneapolis Heart Institute Foundation at Abbott Northwestern Hospital (Minneapolis, Minnesota) who enrolled in one of three clinical trials evaluating CD34^+^ stem cells between 2004 and 2013: a phase I/IIa (NCT00081913), phase II ACT‐34 (NCT00300053; NCT00545610), and phase III RENEW (NCT 01508910). All patients had provided written, informed consent. Enrollment criteria required that patients have Canadian Cardiovascular Society (CCS) class 3‐4 chronic refractory angina despite optimal medical management and be deemed ineligible for further revascularization by percutaneous coronary intervention (PCI) involving stenting or bypass surgery with a minimum of seven anginal episodes per week with limited exercise capacity. All enrolled patients underwent bone marrow cell mobilization with granulocyte colony‐stimulating factor (G‐CSF; 5 μg/kg per day subcutaneously × 4‐5 days; Neupogen; Amgen Inc., Thousand Oaks, California) followed by apheresis on day 5 of mononuclear cells enriched for CD34^+^ cells using a commercially available device.^12^, ^13^, ^14^ Patients were randomly assigned to one of three doses of CD34^+^ cells (1 × 10^4^ cells per kilogram, 1 × 10^5^ cells per kilogram, or 5 × 10^5^ cells per kilogram) or placebo delivered by intramyocardial injections with the NOGA Myostar catheter following electromechanical endocardial mapping using the NOGA system (Biologics Delivery Systems, Diamond Bar, California) to identify ischemic regions of myocardium prior to injection of CD34^+^ cells. A standard of care arm was implemented in the phase III trial only and was not included in this retrospective review.^14^, ^17^ Complete protocols for these trials have been previously described.^12^, ^13^, ^14^, ^17^

Cardiac‐related hospital admissions and cardiovascular procedures were tabulated from electronic medical records for 12 months preceding injection of autologous CD34^+^ cells and for 12 months following injection. Hospital visits for cardiac rhythm abnormalities (including atrial fibrillation, supraventricular tachycardia, ablation procedures, and internal cardiac defibrillator [ICD] changes) were excluded, as were admissions for deep vein thrombosis, enhanced external counterpulsation therapy, and other research studies. Tabulation of hospital visits resulting in coronary procedures included coronary artery bypass grafting, PCI, percutaneous transluminal coronary angioplasty without stenting, coronary angiography, and right heart catheterization. Median total variable costs within the Allina Healthcare system were used to estimate costs of these procedures and any admissions for congestive heart failure or chest pain with and without intervention. Mortality data were obtained from the electronic medical record or from death certificates.

Descriptive statistics include percentage reduction of cardiac‐related emergency department (ED) visits and hospital admissions before and after injection of CD34^+^ cells or placebo, as well as associated hospital costs. A paired *t* test was used to compare number of events and health care expenditures before and after cell therapy. A *P* value of <.05 was considered statistically significant. Because this was an exploratory retrospective analysis with no a priori power calculations performed, the findings of statistical significance should be interpreted with caution.

## 4 RESULTS

A total of 37 patients who received autologous CD34^+^ cells were included in this study. The mean age was 57.9 ± 7.5 years, and 13.5% of patients were female. Baseline characteristics are shown in Table 1. Patients randomized to CD34^+^ cell therapy experienced an average of 1.57 ± 1.39 cardiac‐related hospital visits 12 months before injection compared with 0.78 ± 1.90 cardiac‐related hospital visits 12 months after injection (*P* = .002, Table 2). Overall, the cell therapy group experienced a 50% reduction in hospital visits in the year following CD34^+^ cell therapy.

**TABLE 1:** Baseline characteristics

Characteristics	CD34^+^ cell therapy (n = 37)
Demographics	
Age, mean ± SD, yr	57.9 ± 7.5
Female, %	13.5
Cardiovascular risk factors	
HTN, %	83.8
Smoking status (current or former), %	48.6
Diabetes, %	40.5
Hyperlipidemia, %	97.3
Medical history	
Prior MI, %	48.6
Total CABG surgeries, n^a^	27
Prior PCI procedures, %	100.0
Prior TMR, %	8.1

^a^ 16.2% of subjects (n = 6) underwent multiple CABG surgeries.

Abbreviations: CABG, coronary artery bypass graft; HTN, hypertension; MI, myocardial infarction; PCI, percutaneous coronary intervention; TMR, transmyocardial laser revascularization.

**TABLE 2:** Cardiac‐related hospital visits 12 months before and after injection of CD34^+^ cells (n = 37)

Hospital visits	Before injection	After injection
ED visits		
Chest pain/shortness of breath	7	8
TIA/CVA	0	1
Hospital admits		
Angiography	24	5
CABG surgery	2	1
Chest pain/shortness of breath	7	6
PCI procedure	17	4
PTCA procedure	1	2
TIA/CVA	0	2
Total visits	58	29
Mean visits per patient (±SD), n	1.57 (±1.39)	0.78 (±1.90)

*Note:* Values are n unless otherwise specified.

Abbreviations: CABG, coronary artery bypass graft; CVA, cerebrovascular accident; ED, emergency department; PCI, percutaneous coronary intervention; PTCA, percutaneous transluminal coronary angioplasty; TIA, transient ischemic attack.

When considering interventional coronary procedures only, patients in the CD34^+^ group experienced an average of 1.2 ± 0.91 events (n = 18) 12 months before injection compared with 0.32 ± 0.75 events (n = 6) 12 months after injection (*P* < .0001). Overall, the cell therapy group experienced a 73% reduction in coronary procedures in the 12 months following their injection. The total median variable costs of all cardiac hospital visits and procedures significantly decreased by 62% in the cell therapy group following injection (*P* = .03, Table 3) from $8864 to $3338, resulting in a cost savings of $5500 per subject.

**TABLE 3:** Median total variable costs of all cardiovascular hospital visits and procedures 12 months before and after injection of CD34^+^ cells ($USD, n = 37)

Event	Cost per visit, $USD	Preinjection overall cost, $USD	Postinjection overall cost, $USD
ED visit—chest pain	1000	7000	8000
Hospital admit—chest pain	2000	14 000	12 000
Angiography procedure	3500	84 000	17 500
PTCA procedure	7000	7000	14 000
PCI procedure	8000	136 000	32 000
CABG surgery	40 000	80 000	40 000
Total costs		$328 000	$123 500
Mean cost per patient		$8864	$3337
Mean reduction, %			62 (*P* = .03)

Abbreviations: $USD, United States Dollars; CABG, coronary artery bypass graft; ED, emergency department; PCI, percutaneous coronary intervention; PTCA, percutaneous transluminal coronary angioplasty.

Mortality in the CD34^+^ group since enrollment was significantly less than in the placebo group. The total number of deaths in the CD34^+^ group was nine (24%), compared with eight (47%) in the placebo group. In the CD34^+^ group, five deaths were due to cardiovascular disease, two deaths were due to cancer, and cause of death was unavailable for two subjects. The average time between treatment with CD34^+^ cells and death was 6.5 ± 2.4 years. In contrast, placebo patients died an average of 3.7 ± 1.9 years after enrollment, resulting in a significantly shorter timespan compared with the CD34^+^ group (*P* = .02).

Although not the focus of this investigation, subjects randomized to the placebo group (n = 19) also experienced fewer hospitalizations and procedures in the year following injection, from 1.26 ± 0.87 hospital visits and procedures before injection to 0.63 ± 1.42 hospital visits after injection, although this decline was not significant because of the smaller sample size. However, postinjection placebo subjects did exhibit a significant 70% reduction in their health care expenditures, that translated to a significant cost savings of $3700 per subject, although less than the total cost savings per patient in the CD34^+^ treatment group (Table 4).

**TABLE 4:** Median total variable costs of all cardiovascular hospital visits and procedures 12 months before and after injection of placebo subjects ($USD, n = 19)

Event	Cost per visit, $USD	Preinjection overall cost, $USD	Postinjection overall cost, $USD
ED visit—chest pain	1000	1000	5000
Hospital admit—chest pain	2000	8000	8000
Angiography procedure	3500	42 000	3500
PTCA procedure	7000	7000	7000
PCI procedure	8000	32 000	8000
Admit CHF	6000	12 000	0
Total costs		$102 000	$31 500
Mean cost per patient		$5368	$1658

Abbreviations: $USD, United States Dollars; CHF, congestive heart failure; ED, emergency department; PCI, percutaneous coronary intervention; PTCA, percutaneous transluminal coronary angioplasty.

## 5 DISCUSSION

In the 12 months following intramyocardial injection of CD34^+^ stem cells, significant reductions in cardiac‐related ED visits and hospital admissions, interventional coronary procedures, and associated hospital expenditures were observed compared with the 12 months before injection.

The reduction in hospital visits following CD34^+^ therapy is consistent with results from a recent analysis of a European cell therapy trial^18^ using intramyocardial injections of bone marrow mononuclear cells (BMCs). This nonrandomized study of 100 consecutive patients with refractory angina tracked ED visits and hospital admissions 2 years before and 2 years after intramyocardial injection of BMCs. Following treatment, ED visits and hospital admissions decreased by 50% and 70%, respectively, and coronary angiography and PCI decreased by more than 90%. However, although health care use decreased, no cost analysis was performed. A small Brazilian study^16^ of 10 patients with a median follow‐up time of almost 4 years reported similar reductions in interventional coronary procedures and length of hospital stays following stem cell treatment. However, the study authors noted that the reduction in procedures may have reflected the subjects' ineligibility for further revascularization rather than improvements in coronary artery disease. In contrast, we recently reported that up to 25% of “no‐option” patients with refractory angina will still undergo revascularization less than 2 years following this diagnosis.^19^

Importantly, ours is the first study to document a reduction in mortality in addition to the decrease in health care costs and adverse events with the CD34^+^ cell product and is the only cost study that included a placebo group. We observed that mortality was halved for those patients that received CD34^+^ compared with patients who received placebo. Furthermore, among patients who eventually died, those that received CD34^+^ cells lived twice as long as those who received placebo, supporting a true therapeutic benefit for this therapy.

The decrease in hospital visits among placebo patients after enrollment may reflect a placebo effect or a real benefit of G‐CSF. Alternatively, the process of intramyocardial injection itself may exert an antianginal effect or stimulate neovascularization. However, Hossne et al^16^ found promonocyte and lymphocyte concentrations of BMCs to be positively correlated with CCS improvement at 12 months, which suggests that stem cells play a direct role in the physiological mechanism of angina reduction. The phase II ACT‐34 trial^13^ included in this present study found that patients who received a low‐dose injection of cells (1 × 10^5^ CD34^+^ cells per kilogram body weight) exhibited a significant reduction in angina frequency and a significant improvement in exercise tolerance time at 12 months compared with placebo patients, which was also confirmed in the meta‐analysis.^15^

Given that the long‐term mortality rates among this patient population are relatively low,^20^ a greater emphasis should be placed on improving quality of life. A reduction in resource use is also important as health care costs continue to rise. From 2014 to 2015, the direct and indirect costs of heart disease were estimated at $218.7 billion, and the cost of coronary heart disease is projected to double between 2015 and 2030.^21^ The reduction in hospital visits 1 year following treatment with CD34^+^ cells suggests that cell therapy warrants further investigation as a cost‐effective treatment option for patients with refractory angina. It is also possible that the cost benefit of this therapy demonstrated over the first year will continue to grow over time. Because cell therapy is associated with significant costs of procedures and cell processing, it is hoped that the reductions in expenditures demonstrated with this therapy will justify the eventual insurance coverage of this therapy once Food and Drug Administration approval is obtained.

Our work also supports the observation that placebo‐treated patients accrue substantial benefit from participation in clinical trials, as patients in the placebo group also demonstrated a reduction in cardiac‐related hospital events and costs, although to a lesser degree than the CD34^+^ group. Our findings of no signs of risk or early adverse cardiovascular events or increase in hospitalizations support the contention that the procedures associated with CD34^+^ cell mobilization, apheresis, and intramyocardial injections are safe without an accumulation of risk in the postprocedure period. However, in spite of these benefits, the placebo group demonstrated greater mortality than the CD34^+^ cell therapy group extending over the long‐term follow‐up period.

## CONFLICT OF INTEREST

T.J.P. declared advisory role with Caladrius Biosciences, Blue Rock Therapeutics, and University of Washington; honoraria with Caladrius Biosciences, Blue Rock Therapeutics, and NovoNordisk; and research funding from CSL Behring, Intracellular Therapeutics, Eli Lilly, Merck, and Janssen Pharmaceuticals. D.W.L. declared leadership position and ownership interest with Caladrius Biosciences. L.I.S. declared leadership position with Minneapolis Heart Institute Foundation, Comcast, and Critical Value Consulting & Analytics; research funding from NIH; and ownership interest with Comcast. J.H.T. declared research funding from National Heart, Lung, and Blood Institute. The other authors indicated no conflicts of interest.

## AUTHOR CONTRIBUTIONS

G.L.J., J.H.T.: collection/assembly of data, manuscript writing, final approval of manuscript; T.D.H., T.J.P., D.W.L.: conception and design, data analysis and interpretation, final approval of manuscript; R.F.G., L.I.S., C.E.S.: data analysis and interpretation, final approval of manuscript.

## Data Availability

The data from this study is not available as it is patient protected health information.

## References

[1] Gallone G , Baldetti L , Tzanis G , et al. Refractory angina: from pathophysiology to new therapeutic nonpharmacological technologies. JACC Cardiovasc Interv. 2020;13:1‐19. 31918927 10.1016/j.jcin.2019.08.055

[2] Mannheimer C . The problem of chronic refractory angina. Report from the ESC joint study group on the treatment of refractory angina. Eur Heart J. 2002;23:355‐370. 11846493 10.1053/euhj.2001.2706

[3] Henry TD , Satran D , Jolicoeur EM . Treatment of refractory angina in patients not suitable for revascularization. Nat Rev Cardiol. 2014;11:78‐95. 24366073 10.1038/nrcardio.2013.200

[4] Kones R. Recent advances in the management of chronic stable angina II. Anti‐ischemic therapy, options for refractory angina, risk factor reduction, and revascularization. http://www.dovepress.com/recent-advances-in-the-management-of-chronic-stable-angina-ii-anti-isc-peer-reviewed-article-VHRM. Accessed May 27, 2019. 10.2147/vhrm.s11100 PMC2941787 20859545

[5] Mukherjee D , Bhatt DL , Roe MT , Patel V , Ellis SG . Direct myocardial revascularization and angiogenesis—how many patients might be eligible? Am J Cardiol. 1999;84:598‐600. 10482164 10.1016/s0002-9149(99)00387-2

[6] Andréll P , Ekre O , Grip L , et al. Fatality, morbidity and quality of life in patients with refractory angina pectoris. Int J Cardiol. 2017;147:377‐382. 10.1016/j.ijcard.2009.09.538 19880202

[7] Povsic TJ , Broderick S , Anstrom KJ , et al. Predictors of long‐term clinical endpoints in patients with refractory angina. J Am Heart Assoc. 2015;4:e001287. 25637344 10.1161/JAHA.114.001287 PMC4345862

[8] Losordo DW , Dimmeler S . Therapeutic angiogenesis and vasculogenesis for ischemic disease: part II: cell‐based therapies. Circulation. 2004;109:2692‐2697. 15184293 10.1161/01.CIR.0000128596.49339.05

[9] Kocher AA , Schuster MD , Szabolcs MJ , et al. Neovascularization of ischemic myocardium by human bone‐marrow–derived angioblasts prevents cardiomyocyte apoptosis, reduces remodeling and improves cardiac function. Nat Med. 2001;7:430‐436. 11283669 10.1038/86498

[10] Kawamoto A , Tkebuchava T , Yamaguchi JI , et al. Intramyocardial transplantation of autologous endothelial progenitor cells for therapeutic neovascularization of myocardial ischemia. Circulation. 2003;107:461‐468. 12551872 10.1161/01.cir.0000046450.89986.50

[11] Kawamoto A , Iwasaki H , Kusano K , et al. CD34‐positive cells exhibit increased potency and safety for therapeutic neovascularization after myocardial infarction compared with total mononuclear cells. Circulation. 2006;114:2163‐2169. 17075009 10.1161/CIRCULATIONAHA.106.644518

[12] Losordo DW , Schatz RA , White CJ , et al. Intramyocardial transplantation of autologous CD34^+^ stem cells for intractable angina: a phase I/IIa double‐blind, randomized controlled trial. Circulation. 2007;115:3165‐3172. 17562958 10.1161/CIRCULATIONAHA.106.687376

[13] Losordo DW , Henry TD , Davidson C , et al.; the ACT34‐CMI InvestigatorsIntramyocardial, autologous CD34^+^ cell therapy for refractory angina. Circ Res. 2011;109:428‐436. 21737787 10.1161/CIRCRESAHA.111.245993 PMC3190575

[14] Povsic TJ , Henry TD , Traverse JH , et al.; RENEW InvestigatorsThe RENEW trial. JACC Cardiovasc Interv. 2016;9:1576‐1585. 27491607 10.1016/j.jcin.2016.05.003

[15] Henry TD , Losordo DW , Traverse JH , et al. Autologous CD34^+^ cell therapy improves exercise capacity, angina frequency and reduces mortality in no‐option refractory angina: a patient‐level pooled analysis of randomized double‐blinded trials. Eur Heart J. 2018;39:2208‐2216. 29315376 10.1093/eurheartj/ehx764

[16] Hossne NA , Cruz E , Buffolo E , et al. Long‐term and sustained therapeutic results of a specific promonocyte cell formulation in refractory angina: ReACT® (Refractory Angina Cell Therapy) clinical update and cost‐effective analysis. Cell Transplant. 2015;24:955‐970. 24819720 10.3727/096368914X681595

[17] Povsic TJ , Junge C , Nada A , et al. A phase 3, randomized, double‐blinded, active‐controlled, unblinded standard of care study assessing the efficacy and safety of intramyocardial autologous CD34^+^ cell administration in patients with refractory angina: design of the RENEW study. Am Heart J. 2013;165:854‐861. 23708155 10.1016/j.ahj.2013.03.003

[18] Rodrigo SF , Mann I , van Ramshorst J , et al. Reduction of healthcare utilization after bone marrow cell therapy for refractory angina pectoris. Int J Cardiol. 2016;202:571‐572. 26447664 10.1016/j.ijcard.2015.09.103

[19] Sharma R , Tradewell M , Kohl LP , et al. Revascularization in "no option" patients with refractory angina: frequency, etiology and outcomes. Catheter Cardiovasc Interv. 2018;92:1215‐1219. 30079551 10.1002/ccd.27707

[20] Henry TD , Satran D , Hodges JS , et al. Long‐term survival in patients with refractory angina. Eur Heart J. 2013;34:2683‐2688. 23671156 10.1093/eurheartj/eht165

[21] Benjamin EJ , Muntner P , Alonso A , et al.; American Heart Association Council on Epidemiology and Prevention Statistics Committee and Stroke Statistics SubcommitteeHeart disease and stroke statistics—2019 update: a report from the American Heart Association. Circulation. 2019;139:e56‐e528. 30700139 10.1161/CIR.0000000000000659

